# Global Transcript Profiles of Fat in Monozygotic Twins Discordant for BMI: Pathways behind Acquired Obesity 

**DOI:** 10.1371/journal.pmed.0050051

**Published:** 2008-03-11

**Authors:** Kirsi H Pietiläinen, Jussi Naukkarinen, Aila Rissanen, Juha Saharinen, Pekka Ellonen, Heli Keränen, Anu Suomalainen, Alexandra Götz, Tapani Suortti, Hannele Yki-Järvinen, Matej Orešič, Jaakko Kaprio, Leena Peltonen

**Affiliations:** 1 Obesity Research Unit, Department of Psychiatry, Helsinki University Central Hospital, Helsinki, Finland; 2 Department of Medicine, Division of Diabetes, Helsinki University Central Hospital, Helsinki, Finland; 3 Finnish Twin Cohort Study, Department of Public Health, University of Helsinki, Helsinki, Finland; 4 Department of Molecular Medicine, National Public Health Institute, Helsinki, Finland; 5 Department of Medical Genetics and Research Program of Molecular Medicine, University of Helsinki, Finland; 6 Research Program of Molecular Neurology and Department of Neurology, University of Helsinki and Helsinki University Central Hospital, Helsinki, Finland; 7 VTT Technical Research Centre of Finland, Espoo, Finland; 8 Department of Mental Health and Alcohol Research, National Public Health Institute, Helsinki, Finland; 9 The Wellcome Trust Sanger Institute, Cambridge, United Kingdom; 10 Broad Institute, Massachusetts Institute of Technology, Cambridge, Massachusetts, United States of America; Lund University, Sweden

## Abstract

**Background:**

The acquired component of complex traits is difficult to dissect in humans. Obesity represents such a trait, in which the metabolic and molecular consequences emerge from complex interactions of genes and environment. With the substantial morbidity associated with obesity, a deeper understanding of the concurrent metabolic changes is of considerable importance. The goal of this study was to investigate this important acquired component and expose obesity-induced changes in biological pathways in an identical genetic background.

**Methods and Findings:**

We used a special study design of “clonal controls,” rare monozygotic twins discordant for obesity identified through a national registry of 2,453 young, healthy twin pairs. A total of 14 pairs were studied (eight male, six female; white), with a mean ± standard deviation (SD) age 25.8 ± 1.4 y and a body mass index (BMI) difference 5.2 ± 1.8 kg/m^2^. Sequence analyses of mitochondrial DNA (mtDNA) in subcutaneous fat and peripheral leukocytes revealed no aberrant heteroplasmy between the co-twins. However, mtDNA copy number was reduced by 47% in the obese co-twin's fat. In addition, novel pathway analyses of the adipose tissue transcription profiles exposed significant down-regulation of mitochondrial branched-chain amino acid (BCAA) catabolism (*p* < 0.0001). In line with this finding, serum levels of insulin secretion-enhancing BCAAs were increased in obese male co-twins (9% increase, *p* = 0.025). Lending clinical relevance to the findings, in both sexes the observed aberrations in mitochondrial amino acid metabolism pathways in fat correlated closely with liver fat accumulation, insulin resistance, and hyperinsulinemia, early aberrations of acquired obesity in these healthy young adults.

**Conclusions:**

Our findings emphasize a substantial role of mitochondrial energy- and amino acid metabolism in obesity and development of insulin resistance.

## Abstract 2

Leena Peltonen and colleagues uncover the metabolic changes that result from obesity through an analysis of genetically identical twin pairs in which one was obese and the other was not.

## Editors' Summary

**Background.:**

Around the world, the proportion of people who are obese (people with an unhealthy amount of body fat) is increasing. In the US, for example, 1 adult in 7 was obese in the mid 1970s. That is, their body mass index (BMI)—their weight in kilograms divided by their height in meters squared—was more than 30. Nowadays, 1 US adult in 3 has a BMI this high and, by 2025, it is predicted that 1 in 2 will be obese. This obesity epidemic is being driven by lifestyle changes that encourage the over-consumption of energy-rich foods and discourage regular physical activity. The resultant energy imbalance leads to weight gain (the excess energy is stored as body fat or adipose tissue) and also triggers numerous metabolic changes, alterations in the chemical processes that convert food into the energy and various substances needed to support life. These obesity-related metabolic changes increase a person's risk of developing adverse health conditions such as diabetes, a condition in which dangerously high levels of sugar from food accumulate in the blood.

**Why Was This Study Done?:**

The changes in human fat in obesity have not been completely understood, although the abnormal metabolism of adipose tissue is increasingly seen as playing a critical part in excessive weight gain. It has been very difficult to decipher which molecular and metabolic changes associated with obesity are the result of becoming obese, and which might contribute towards the acquisition of obesity in humans in the first place. To discover more about the influence of environment on obesity-induced metabolic changes, the researchers in this study have investigated these changes in pairs of genetically identical twins.

**What Did the Researchers Do and Find?:**

The researchers recruited 14 pairs of genetically identical Finnish twins born between 1975 and 1979 who were “obesity discordant”—that is, one twin of each pair had a BMI of about 25 (not obese); the other had a BMI of about 30 (obese). The researchers took fat and blood samples from each twin, determined the insulin sensitivity of each, and measured the body composition and various fat stores of each. They found that the obese twins had more subcutaneous, intra-abdominal, and liver fat and were less insulin sensitive than the non-obese twins. Insulin sensitivity correlated with the amount of liver fat. Analysis of gene expression in the fat samples showed that 19 gene pathways (mainly inflammatory pathways) were expressed more strongly (up-regulated) in the obese twins than the non-obese twins, whereas seven pathways were down-regulated. The most highly down-regulated pathway was a mitochondrial pathway involved in amino acid breakdown, but mitochondrial energy metabolism pathways were also down-regulated. Finally, mitochondrial DNA copy number in fat was reduced in the obese twins by nearly half, a novel observation that could partly account for the obesity-induced metabolic defects of these individuals.

**What Do These Findings Mean?:**

These and other findings identify several pathways that are involved in the development of obesity and insulin resistance. In particular, they suggest that changes in mitochondrial energy production pathways and in mitochondrial amino acid metabolism pathways could play important roles in the development of obesity and of insulin resistance and in the accumulation of liver fat even in young obese people. The study design involving identical twins has here produced some evidence for aberrations in molecules critical for acquired obesity. The results suggest that careful management of obesity by lifestyle changes has the potential to correct the obesity-related metabolic changes in fat that would otherwise lead to diabetes and other adverse health conditions in obese individuals. In addition, they suggest that the development of therapies designed to correct mitochondrial metabolism might help to reduce the illnesses associated with obesity.

**Additional Information.:**

Please access these Web sites via the online version of this summary at http://dx.doi.org/10.1371/journal.pmed.0050051.

- The MedlinePlus encyclopedia has pages on obesity and diabetes (in English and Spanish)
- The US Centers for Disease Control and Prevention provides information on all aspects of obesity (in English and Spanish)
- The UK National Health Service's health Web site (NHS Direct) provides information about obesity
- The International Obesity Taskforce provides information about preventing obesity and on diabetes and obesity
- The UK Foods Standards Agency and the United States Department of Agriculture provide online tools and useful advice about healthy eating for adults and children
- Information is available for patients and carers from the US National Diabetes Information Clearinghouse on  diabetes, including information on insulin resistance

## Introduction

Adipose tissue is a key player in obesity-related metabolic dysfunctions, and is closely linked to other peripheral organs that control energy flux [1]. To understand the pathogenesis of adipose tissue dysfunction at a molecular level, a global view of the networks and their interactions should be more informative than monitoring single-gene effects [2]. Expression array studies on human adipose tissue have shown that the various inflammatory pathways are activated in obesity [3,4] and are down-regulated following weight loss [5]. The major inflammatory cells in fat are macrophages of different subtypes [6,7], which scavenge adipocyte debris from necrotic cells [8]. Paradoxically in obesity, macrophage-secreted factors may result in impaired adipogenesis [9] by inhibiting the expression of adipogenic transcription factors including peroxisome proliferator-activated receptor-gamma (PPARγ) [10]. Impaired adipogenesis and defects in mitochondrial energy metabolism in subcutaneous adipose tissue have been suggested to shift lipid storage into ectopic insulin-sensitive tissues such as the liver, skeletal muscle, and pancreas, resulting in severe insulin resistance [11,12]. Induction of adipocyte differentiation [13] and mitochondrial biogenesis [14] in subcutaneous sites could reverse these abnormalities [15]. However, molecular pathways related to aberrations in adipocyte differentiation in obesity remain unknown. Furthermore, although mitochondrial dysfunction has been previously identified in muscle in human obesity and type 2 diabetes [16–18] as well as in the adipocytes of obese rodents [19,20], direct evidence of possible defects in mitochondrial function in adipocytes in human obesity is lacking.

Obesity-related gene expression variations in fat are influenced by complex interactions of genes and the environment, the contributions of which are difficult to disentangle. Studying tissue samples of rare monozygotic (MZ) twin pairs discordant for body weight provides a unique opportunity to explore the effects of acquired obesity, since these individuals share not only identical genetic background, but also early life events and family environment. In this study, we utilized a special collection of such weight-discordant MZ twins with identical genetic background and through in-depth phenotyping and analysis of their global gene expression profiles in adipose tissue, aimed at describing the changes in biological pathways that accompany acquired obesity and relate these to the associated clinically relevant aberrations.

## Methods

### Participants

The participants were recruited from a population-based longitudinal study of five consecutive birth cohorts (1975–1979) of twins, their siblings, and their parents (*n* = 2,453 families), identified through the national population registry of Finland [21]. Twin pairs included in the current study were enrolled on the basis of their responses to questions on weight and height at age 23–27 y. From this cohort, we searched for the top 5% most obesity-discordant MZ twin pairs (one co-twin not obese [BMI ∼25 kg/m^2^], and the other one obese [BMI ∼30 kg/m^2^]), with no significant height differences (<4 cm). After screening all MZ twin pairs (*n* = 658), we identified 18 pairs above the 95th percentile of BMI differences (3.1 kg/m^2^) [22–27]. Of these pairs, 14 (eight male and six female pairs) were willing to participate, and 13 pairs (eight male and five female pairs, BMI differences 3.3–10.0 kg/m^2^, height differences <3 cm) had adipose tissue samples available for the present study. All pairs were researcher-designated white, and their mean age was 25.8 ± 1.4 y. The participants were healthy (based on medical history, clinical examination, and structured psychiatric interview), normotensive, and did not use any medications except oral contraceptives. Their weights had been stable for at least 3 mo prior to the study. Females were scheduled to attend during the follicular phase of their menstrual cycle. Monozygosity was confirmed by genotyping of ten informative genetic markers [23]. The participants provided written informed consent. The protocol was designed and performed according to the principles of the Helsinki Declaration and was approved by the Ethical Committee of the Helsinki University Central Hospital.

### Study Design

**Figure 1:**
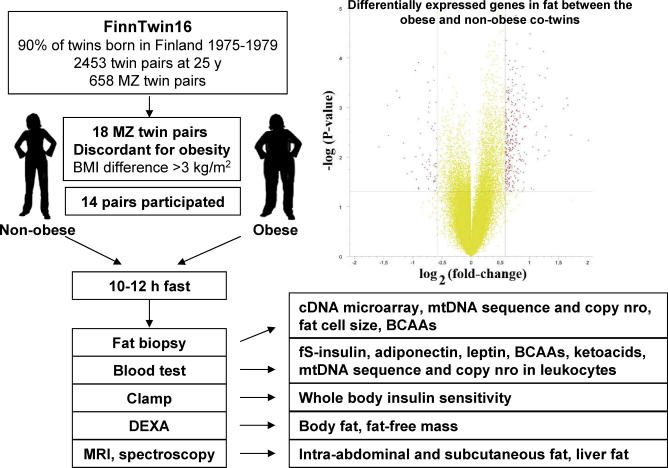
Study Design and the Global Transcript Profiles in Subcutaneous Fat in the Co-twins

The study design is shown in Figure 1. A more detailed description of methods is provided in Text S1. In brief, all participants were studied starting at 8 a.m. after an overnight fast. Venous blood samples were obtained and plasma with EDTA and serum were separated by centrifugation and stored at −80 °C until the analyses of insulin, adiponectin, leptin, high-sensitivity CRP, leucine, valine, and isoleucine, and a leucine breakdown product, α-ketoisocaproate. Whole-body insulin sensitivity (the M-value, measured in mg·kg fat-free mass^−1^·min^−1^) was measured using the euglycemic–hyperinsulinemic clamp technique [28]. Body composition was measured by dual-energy x-ray absorptiometry (DEXA), subcutaneous and intra-abdominal fat by MRI of 16 transaxial scans reaching from 8 cm above to 8 cm below the fourth and fifth lumbar interspace, and liver fat content by proton spectroscopy [22].

Fat cell size was determined from fresh subcutaneous abdominal adipose tissue samples treated with collagenase. RNA was prepared from frozen fat and used for the global gene expression analyses in Affymetrix U133 Plus 2.0 chips. Affymetrix gene expression chip data were analyzed using MAS-5 according to the manufacturer's recommendations, and preprocessed with the GC-RMA algorithm. Given the unique setup involving MZ twins, the expression data were further “co-twin normalized,” which involved dividing the obese twin's expression values with those of the non-obese co-twin in order to correct for the identical genetic background. The pathway analysis was done with an in-house nonparametric analysis algorithm utilizing the Gene Ontology (GO) categories (previously used in [29–32]), in which the objective is to find the optimal regulated pathway compositions without a priori criteria for significance for individual genes' significant regulation or arbitrary *p*-value/fold change cut-offs. The method uses an iterative cumulative hypergeometric distribution *p*-value–based calculation, and empirical *p*-values reported are interpreted from the distribution of 10,000 permutation cycles as described in Text S1. As a feature of the tree-like structure of the GO classifications, gene sets get progressively larger and less descriptive when moving down the tree. Therefore, in order to concentrate on the biologically more meaningful pathway collections, an arbitrary cut-off of a maximum of 250 genes was chosen as the largest reported gene list.

MtDNA copy number in fat and blood leukocytes was normalized against a nuclear single-copy gene, by amplification of the *cytB* gene from mtDNA and *APP* from nuclear DNA, from total DNA extracted from adipose tissue and leukocyte samples. Real-time PCR with TaqMan probes was utilized, and quantitation was performed in the exponential amplification phase. The full mitochondrial DNA sequences were determined from extracted total DNAs by high-throughput mtDNA sequence analyses utilizing amplification of mtDNA in two large fragments, followed by DNA sequencing with several internal primers, as specified in Text S1.

### Statistical Analyses

The statistical analyses of the clinical parameters were performed using Stata statistical software (release 8.0; Stata Corporation). Between–co-twin comparisons were made by paired Wilcoxon signed ranks test. Male and female pairs were combined because, by definition, MZ co-twins are matched for gender. Within pairs, Spearman correlation was used to test the effects of the extent of BMI discordance on gene expression pathways. In individual twins, Pearson correlation and multiple regression analyses, corrected for clustered sampling of co-twins by survey methods [33], were performed to determine the relationships between gene expression and clinical characteristics. The survey commands in Stata require the use of parametric tests. Log-transformation was used to normalize the distribution of liver fat and serum adiponectin.

## Results

### Clinical Characteristics of the Obesity-Discordant Monozygotic Co-twins

The obese co-twins of the pairs discordant for obesity were on average 15.2 kg (20%) heavier than the non-obese co-twins (Table 1). This weight discordance had developed in postpubertal adolescence and thus represented early obesity [23]. While a marginal difference in birth weight could be observed, this had no correlation with any adult measures of obesity. Dietary intake of energy, protein, carbohydrate, or total fat did not differ between the co-twins, but the obese co-twins consumed less mono- and polyunsaturated fatty acids [22]. Physical activity at the time of the study and in late adolescence was lower in the obese co-twins [34]. Using detailed body composition measurements (DEXA, MRI, and spectroscopy), we could define the difference in total body fat and its distribution in these twin pairs. The obese co-twins had more fat subcutaneously, intra-abdominally, and in the liver (Table 1). They also had significantly larger fat cells. The obese co-twins' whole body insulin sensitivity (the M-value) and fasting serum adiponectin concentrations were significantly lower and insulin concentrations higher than those of their co-twins. The differences within the pairs were similar for males and females. All measurements of insulin sensitivity correlated closely with liver fat in the individual twins (M-value *r* = −0.65, *p* = 0.001; adiponectin *r* = −0.50, *p* = 0.009; and fasting serum (fS)-insulin *r* = 0.65, *p* = 0.001). The correlations between insulin sensitivity and subcutaneous and intra-abdominal fat were slightly lower (e.g., the correlation between fS-insulin and subcutaneous fat was *r* = 0.45, *p* = 0.016 and that between fS-insulin and intra-abdominal fat *r* = 0.47, *p* = 0.013.).

**Table 1:**
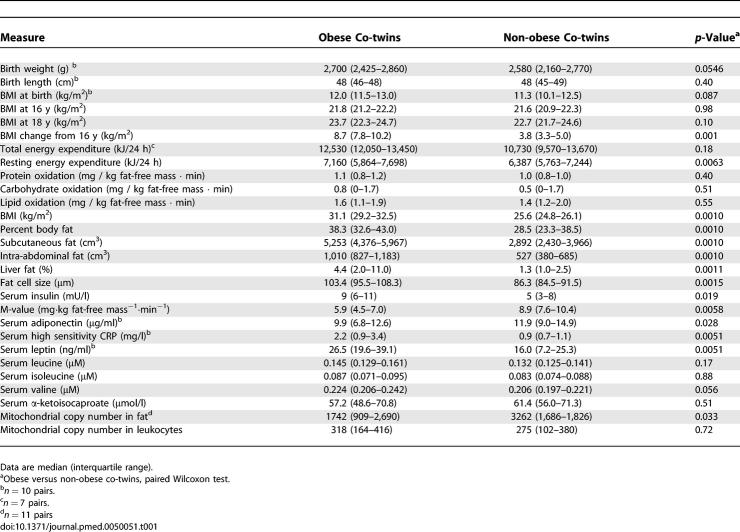
Physical and Biochemical Characteristics of the Obesity-Discordant Monozygotic Twin Pairs (*n* = 14)

### Transcript Profiles Reveal Significant Changes in the Obese Co-twins

Recent studies integrating expression profiling with linkage analysis have shown that the single most important determinant for global transcript profiles in rodent fat is gender [35]. This pattern was also evident in our non-supervised clustering analyses, in which the transcription profiles of the fat biopsies were clustered according to gender (unpublished data). The method we used to normalize the global expression values of each MZ twin to their co-twin (twin normalization) not only eliminated the genetic component, but also corrected for between-gender differences of the variance in transcript levels [36]. Following normalization, the samples no longer exhibited clustering by gender.

The volcano plot in Figure 1 shows many similarities but also significant differences in the transcription profiles between the obese and non-obese twins. We used here an in-house–designed nonparametric analysis algorithm for discovering more subtly regulated pathways. The Gene Ontology (GO) classifications were used to explore the pathways that were significantly different between the obese and non-obese co-twins. After pathways with more than 250 genes were filtered off, 19 up-regulated (Tables 2 and S1) and seven down-regulated pathways (Tables 3 and S2) were identified in the obese co-twins.

**Table 2:**
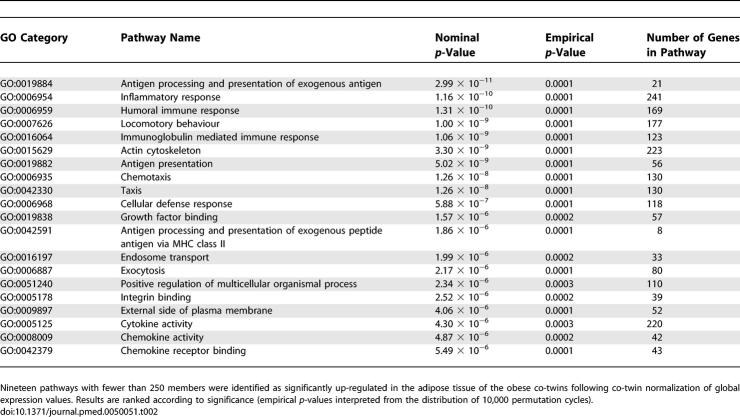
Pathways Up-regulated in the Obese Co-twins

**Table 3:**
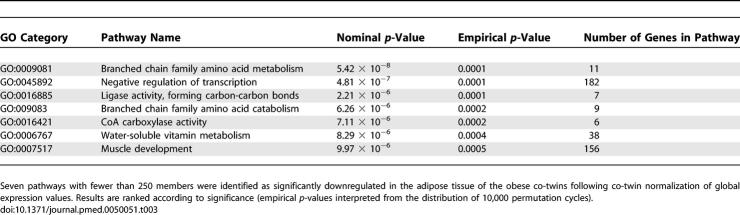
Pathways Down-regulated in the Obese Co-twins

### Inflammation and Cytoskeleton Pathways Up-regulated in the Fat of Obese Co-twins

Of the 19 up-regulated pathways, 15 were related to inflammation and the rest involved organization of the cytoskeleton (actin cytoskeleton, nominal/empirical *p* = 3.3 × 10^−9^/<1.0· × 10^−4^), cell growth (growth factor binding, *p* = 1.6 × 10^−6^/2.0 × 10^−5^), and intracellular transport (endosome transport, *p* = 2.0 × 10^−6^/2.0 × 10^−5^ and exocytosis, *p* = 2.2 × 10^−6^/1.0 × 10^−5^) (Tables 2 and S1). Both innate and adaptive immune systems showed activation in the obesity-induced inflammation cascades. Several cytokines specific for innate immune cells, mainly macrophages (e.g., *MCP-1, MCP-2, MIP-1alpha-R, MIP-4, MIR-10*) but also dendritic (*CLEC7A, TNFSF13B*) and killer cells (*KLRB1*) were present in the up-regulated pathways. The complement system, both the components of the classic pathway (complement C1 [*C1S, C1QB, C1QC*] and C3a [*C3AR1*]) and the alternative pathway (C3/C5 convertase [*CFB*]) were up-regulated in the obese co-twins. An overexpression of MHC class II molecules was also observed, providing some basis for a hypothesis that recognition of antigens by lymphocytes (cells of the adaptive immunity), would be involved in obesity. T cell-specific cytokines (*CCL5, CXCL9*) and their receptors (*IL1R2, IL1RN*) were found in the up-regulated pathways as well as a few signs of B cell activation (*TNFSF13B*).

The most overexpressed gene (5.9-fold) in the obese co-twins was osteopontin (*SPP1*), a Th1 cytokine, which is involved in macrophage recruitment and stimulation of T cell proliferation during inflammation [37]. It was a constituent of seven different inflammatory pathways in the hierarchically clustered GO tree (Table S1). Expression of *SPP1* was significantly correlated with liver fat (*r* = 0.42) and fS-insulin (*r* = 0.43) (both *p* < 0.05), suggesting a role in the pathogenesis of insulin resistance syndrome, including nonalcoholic fatty liver disease.

The average activity of the regulated part of the whole pathway, expressed as the mean centroid (Text S1) [16], was used to test for correlations between the pathway “activity” and clinical characteristics. Inflammation pathways correlated closely with measures of adiposity, insulin resistance, and low-grade inflammation, e.g., the inflammatory response pathway correlated with subcutaneous (*r* = 0.57), intra-abdominal (*r* = 0.61), and liver fat (*r* = 0.60) (all *p* ≤ 0.002); the M-value (*r* = −0.45); adiponectin (*r* = −0.56); fS-insulin (*r* = 0.56); and high-sensitivity C-reactive protein (CRP) (*r* = 0.38) (all *p* ≤ 0.01).

### Mitochondrial Branched Chain Family Amino Acid Catabolism Down-regulated in the Fat of Obese Co-twins

**Figure 2:**
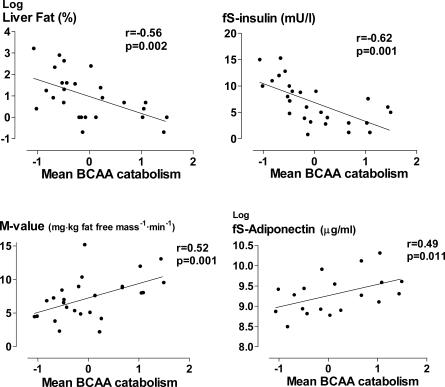
The Relationships between the Mean Expression of the BCAA catabolism Pathway and Liver Fat, fS-Insulin, Whole Body Insulin Sensitivity (the M-value), and fS-Adiponectin

The most significantly down-regulated pathway in the obese co-twins was branched chain family amino acid (BCAA) catabolism (nominal/empirical *p* = 5.4 × 10^−8^/<1.0 × 10^−4^), mostly contributed by the genes encoding the mitochondrial components of the pathway (Tables 3 and S2). Both genes common to the degradation of all BCAAs, i.e., leucine, isoleucine and valine (*BCAT2, BCKDHB*) and those specific for the degradation of leucine (*MCCC1, MCCC2, AUH*) and valine (*HIBADH, ALDH6A1, ACAD8*), were down-regulated in the obese co-twins. The mean centroid of the BCAA catabolism pathway correlated negatively with all measures of body fat, i.e., with liver fat (*r* = −0.56, *p* = 0.002) (Figure 2), subcutaneous fat (*r* = −0.49, *p* = 0.01), and intra-abdominal fat (*r* = −0.47, *p* = 0.01). In a multiple regression analysis including these fat depots, only liver fat remained significantly associated with BCAA catabolism (*R* ^2^ = 29%). Further underscoring its importance, “BCAA catabolism” remained significant (*β* = −0.38, *p* = 0.018) even after including the four most up- and down-regulated pathways (from Tables 2 and 3) to explain liver fat in a multiple regression (*R* ^2^ = 63%, *p* = 0.0008).

Decreased mitochondrial BCAA catabolism was associated with insulin resistance (correlation of “BCAA catabolism” with fS-insulin *r* = −0.62, *p* = 0.001, M-value *r* = 0.52, *p* = 0.001, and S-adiponectin *r* = 0.49, *p* = 0.011, Figure 2) and low-grade inflammation (high-sensitivity CRP *r* = −0.41, *p* = 0.005). To provide some evidence for the biological significance of this finding at the whole body level, we measured serum concentrations of a leucine breakdown product, α-ketoisocaproate, in plasma and confirmed that lowered BCAA catabolism activity was paralleled by decreased α-ketoisocaproate levels (*r* = 0.44, *p* = 0.027). Furthermore, we found in males that serum leucine and valine as well as total BCAA (leucine, valine, isoleucine) concentrations were higher in the plasma of obese than non-obese co-twins (median total BCAA 0.47 μM versus. 0.43 μM, *p* = 0.025) and correlated significantly with fS-insulin (total BCAA and insulin *r* = 0.50, *p* = 0.028). This result is consistent with earlier studies describing high serum BCAAs in obesity [38] and hyperinsulinemia [39]. These correlations were not found at a statistically significant level in female co-twins.

### Energy Metabolism and Cell Differentiation Down-regulated in the Obese Co-twins

Acquired obesity in twins was associated with a significant down-regulation of the genes encoding the rate-limiting enzymes in fatty acid synthesis and β-oxidation (Table S2), and especially interesting was the down-regulation of mitochondrial *acetyl-coenzyme A carboxylase beta* (*ACACB*), an important regulator of fatty acid oxidation. Lowering of its transcription was associated with liver fat accumulation (*r* = −0.55, *p* = 0.009). Pyruvate carboxylase down-regulation suggests lowered synthesis of acetyl-CoA. Down-regulation of the genes encoding enzymes essential for NAD(P) synthesis (*NMNAT2, NMNAT3, visfatin*) was associated with down-regulation of NAD-dependent transcription regulators, such as *SIRT1, 3,* and *7* histone deacetylases (*HDAC5, SIN3A*), induction of which is linked to calorie restriction and longevity [40]. Fatty acid synthesis, acetyl-CoA production, NAD(P) biosynthesis, and BCAA catabolism have been found to be up-regulated during the differentiation of 3T3-L1 adipocytes [41]. Our opposite findings would suggest that adipocyte differentiation is impaired in acquired obesity, a finding endorsed by reduced *PPARG* expression in the obese co-twins.

We also saw in our study sample that enlargement of fat cell size, an indirect sign of poor differentiation, was associated with down-regulation of the pathways for negative regulation of transcription (*r* = −0.60), water-soluble vitamin metabolism (*r* = −0.59), and muscle development (*r* = −0.62) (all *p* < 0.001), which involve several differentiation-enhancing factors. Within twin pairs, increased effects were seen on these pathways in twins with greater obesity discordance, but they were not all statistically significant. The larger the BMI discordance, the greater the down-regulation of BCAA catabolism (*r* = −0.42, *p* = 0.16), water-soluble vitamin metabolism (*r* = −0.64, *p* = 0.018), and muscle development (*r* = −0.73, *p* = 0.0050). For other pathways, similar, but nonsignificant results were observed. Furthermore, the greater the increase in the BMI discordance from age 16 to young adulthood, the larger the within-pair down-regulation of, e.g., the BCAA catabolism in fat (*r* = −0.62, *p* = 0.025).

### Mitochondrial DNA Copy Number in Fat Decreased in the Obese Co-twins

The mtDNA sequences of fat showed no evidence for heteroplasmy in co-twins, nor potentially obesity-associated sequence changes between obese and non-obese co-twins in fat or in leukocytes (Figure S1). However, the obese co-twins had a significant reduction of their fat mtDNA copy number, to 53% of that in the non-obese co-twins (*p* = 0.033, Table 1). Confirming the effects of acquired obesity on lowering of fat mtDNA copy number, we analyzed the mtDNA copy number from seven MZ twin pairs concordant for body weight (median BMI for twin A 24.6 kg/m^2^ versus twin B 25.4 kg/m^2^). Their median mtDNA copy numbers were similar, 2,077 versus 2,041, *p* = 0.87. The mtDNA copy numbers in leukocytes were similar in all twin groups.

Amino acid synthesis is linked to energy metabolism, and decreased BCAA catabolism could reflect reduced oxidative energy production caused by low mtDNA. However, mtDNA copy number (*p* > 0.1) did not solely explain variations in liver fat, fS-insulin and the M value in multiple regression analyses where BCAA catabolism (*p* < 0.01) was used as the other explanatory variable. This inability of mtDNA loss to explain in full the decreased catabolism of BCAAs suggests that the metabolic defects of fat related to acquired obesity would not be solely attributed to the reduced number of mtDNA copies. However, this apparent mitochondrial defect is likely to initiate multiple signaling pathways, some critical ones likely involved in BCAA metabolism.

### Longitudinal Data on an Obese Male Twin

While this study design was designed to be purely cross-sectional, we obtained a second measurement of one of the obese male twins, who had gained 10.7 kg (from 93.2 to 103.9 kg) at a re-visit after 3.3 y. We found further pathological changes that were in line with those observed in the whole dataset between all the co-twins. The BMI of this obese twin increased from 28.4 to 32.0 kg/m^2^, with a concomitant increase in all other body fat parameters. Most notable was the 4-fold increase in liver fat (from 2.5% to 10.4%). The M-value describing insulin sensitivity decreased from 7.3 to 3.8 mg·kg fat-free mass^−1^·min^−1^, and serum insulin increased from 12 to 15.3 mU/l. Inflammatory pathways of fat were more up-regulated in the advanced state of obesity, e.g., the mean centroid of the inflammatory response pathway increased 70%. Mitochondrial DNA copy number in fat decreased by 44% and serum BCAA concentrations increased from 0.49 to 0.55 μM and those of leucine from 0.14 to 0.17 μM.

## Discussion

**Figure 3:**
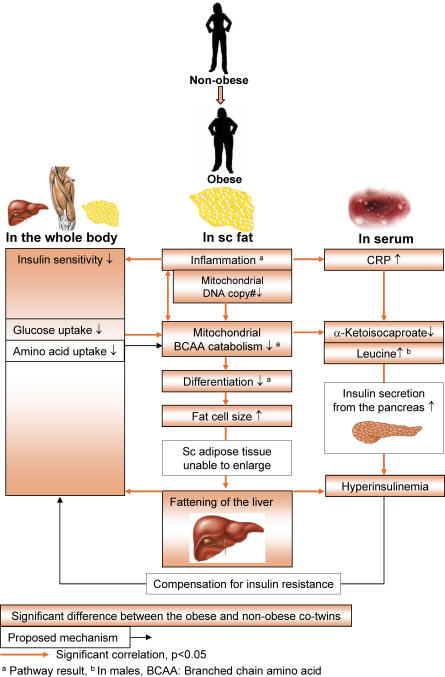
Summary of Findings and Postulated Mechanisms and How They Associate with the Clinical Progression of Obesity-Related Pathologies Obesity already in its early stages in healthy young adults is characterized by marked inflammation of adipose tissue, significantly reduced copy number of mitochondrial DNA, and disturbed mitochondrial energy metabolism—most importantly decreased BCAA catabolism. Down-regulation of mitochondrial energy metabolism and BCAA catabolism is related to impaired differentiation and storage capacity of subcutaneous fat. Fat deposition to the liver increases, which is associated with insulin resistance and hyperinsulinemia. Insulin resistance may impair BCAA catabolism through decreased uptake of amino acids to cells and, as a feedback mechanism, serum BCAA concentrations increase and induce pancreatic insulin secretion. Inflammation may also cause insulin resistance through other mechanism, such as direct action of cytokines or other mediators.

Our carefully phenotyped MZ twins most discordant for obesity represent an ideal model to explore the effects of acquired human obesity independent of genetic factors. By constructing networks of global transcript profiles in subcutaneous fat biopsies, we identified several fundamental pathways with high relevance for obesity and its related insulin resistance (Figure 3). Our data suggest that obesity that is already in its early stages in healthy young adults is characterized by marked inflammation of adipose tissue, significantly reduced mitochondrial DNA copy number, and disturbed mitochondrial energy metabolism—statistically most significantly, the decreased catabolism of insulin secretion–enhancing BCAAs. These impairments correlated with the critical clinical measures of obesity: liver fat accumulation, reduced whole-body insulin sensitivity, hyperinsulinemia, and hypoadiponectinemia.

The observed impairments in adipose tissue function could be associated with insulin resistance by at least the following mechanisms: (1) Inflammation in fat and a direct action of cytokines or other mediators on the insulin signaling cascade [42], (2) decline in mitochondrial DNA content or function [43], and (3) diminished adipocyte differentiation within subcutaneous adipose tissue and subsequent increase in ectopic fat deposition [11,12].

A wide range of inflammatory cascades, both humoral and cellular mediators, seemed to be activated in the obese co-twins' fat. In line with previous studies [6], the major over-expressed innate inflammatory component was related to macrophages. These cells act as scavengers phagocytosing adipose debris [8] and as antigen-presenting cells activating the adaptive immune system. Another up-regulated pathway in obese co-twins involved antigen presentation via MHC class II molecules. This result, together with signs of activation of lymphocytes and the complement system, suggest a widespread induction of previously unrecognized dimensions of the inflammatory response in obesity. In addition, *complement component 3a receptor 1* (*C3ar1*), up-regulated in the obese co-twins, is among the three new genes with causal relationships for obesity in an elegant study in rodents integrating gene expression and DNA variation [44].

A whole range of local and humoral mediators are known to inhibit insulin signaling [42]. Additionally, recent studies show that specific proinflammatory lipid molecules, the lysophosphatidylcholines (LPCs), may impair insulin signaling at the level of insulin receptor substrate-1 (IRS1) and Akt/protein kinase C [45]. LPCs, major components of oxidized low-density lipoprotein (LDL), are phospholipase A2-generated hydrolysis products of phosphatidylcholines, which are connected with the up-regulation of LDL-associated phospholipase A2 (*PLA2G7*) observed in the fat of the obese twins in this study. Furthermore, LPCs activate RhoA GTPases [46], MCP1, and other chemokines [47], which were also up-regulated in this study's obese co-twins. In our previous study, LPCs were over-represented in the serum lipidomic assays in the obese co-twins, and were associated with lower insulin sensitivity [48]. This observation supports the view that LPCs and possibly other lysophospholipids may act as triggers of inflammation in adipose tissue and play a role in obesity-related insulin resistance.

Our finding that the most over-expressed (5.9-fold) gene in the adipose tissue of the obese co-twins was the inflammatory cytokine osteopontin (*SPP1*, also termed *OPN*), involved in the recruitment of macrophages, is of great interest. During the review process of this article, Nomiyama and colleagues published their findings (in a mouse model of diet-induced obesity) that for the first time linked SPP1 to obesity and insulin resistance [49]. Their results suggest that SPP1 plays a key role in the recruitment of macrophages into the adipose tissue in obese mice and thus in the development of insulin resistance. Secretion of SPP1 was increased during obesity and, unlike in wild-type mice, animals lacking SPP1 were protected from developing insulin resistance despite diet-induced obesity. The authors also showed that *SPP1* expression decreased during the differentiation of 3T3-L1 preadipocytes, suggesting that alongside the decrease in *PPARγ* expression observed here, the dramatic increase in *SPP1* expression is yet another marker for poor differentiation of adipocytes in obesity. The over-expression of *SPP1* in obese adipose tissue—here to our knowledge reported for the first time in humans—is an important observation in understanding the development of insulin resistance associated with obesity.

We found considerable depletion of mtDNA in adipose tissue of obese co-twins; to our knowledge this is the first finding of its kind in humans. Such a decline in the mtDNA content of fat cells may decrease the maximal capacity for oxidative phosphorylation, i.e., utilization of fat for energy production. Our study does not yet answer the question of whether mtDNA depletion is a primary event in acquired obesity or whether it is secondary and reactive to, for example, inflammation. However, such a remarkable decrease in mtDNA copy number would have a considerable impact on energy metabolism of fat tissue. Supporting our findings, patients with mutations in polymerase gamma, the mtDNA replicative polymerase, show partial mtDNA depletion and obesity [50]. Furthermore, in our weight-concordant control MZ pairs, the mtDNA copy number levels were nearly identical. Taken together, these results strongly suggest that mtDNA depletion in our study's obese co-twins is weight-dependent. Longitudinal data accessible to us for one obese male further supported this suggestion: after this twin's weight increased by 10 kg within 3 y, mtDNA copy number in his fat tissue declined by 44%.

MtDNA copy number is under nuclear control and can be regulated by proteins of mtDNA maintenance and by cytoplasmic nucleoside pools. Since MZ twins have identical nuclear genes, the depletion of mtDNA we saw in obese co-twins suggests that mtDNA levels in the adipose tissue respond to environmental factors, such as nutrition and exercise. Such an effect has been documented in human skeletal muscle [51]. Genetic factors [18] and age [52] may also influence mtDNA levels and capacity for oxidative energy production. However, controversy remains over whether low mitochondrial oxidative capacity can be accounted for by reduced overall mitochondrial mass or by mitochondrial dysfunction [53,54]. The role of mtDNA levels and mitochondrial dysfunction in obesity has not, to our knowledge, been shown in human adipose tissue before. Here we demonstrate that acquired obesity, at an early stage and independent of genetic variation or age, is associated with significant decreases in mtDNA copy number in fat, together with low expression of transcripts involved in mitochondrial oxidative energy metabolism and BCAA catabolism.

Observations in animal models of obesity (*ob/ob* mouse) and type 2 diabetes (*db/db* mouse) have recently shown a reduction in the mtDNA content of white adipocytes concomitant with smaller mitochondrial size, disturbed mitochondrial morphology, and reduced respiration rates [55]. This pattern was observed only in adipose tissue, not in skeletal muscle or liver. In another study, treatment with the insulin sensitivity-enhancing drug rosiglitazone reversed the dysmorphologic features of *ob/ob* mouse mitochondria and mitochondrial protein profile [19]. These observations emphasize the role of adipose tissue, and especially of the associated mitochondria, in the development of insulin resistance and other obesity-associated pathologies.

Down-regulation of BCAA catabolism in subcutaneous adipose tissue was associated with liver fat accumulation, the mechanisms of which may involve not only reduced mitochondrial respiration in the existing adipocytes, but also reduced differentiation and formation of new adipocytes within the subcutaneous sites, which shifts lipid storage into peripheral tissues. BCAA catabolism is normally activated during adipocyte differentiation, together with the key transcription factor PPARγ and molecules of oxidative energy production (acetyl-CoA utilization and NAD(P) biosynthesis) [41], all of which were coordinately down-regulated in our study's obese co-twins. Impaired adipogenesis, together with impaired mitochondrial biogenesis in subcutaneous adipose tissue, could connect obesity to liver fat accumulation and to the development of type 2 diabetes [11,12].

Following the clue provided by the transcript profiles in fat, we searched for signs of the systemic effects of low adipose tissue mitochondrial BCAA catabolism. The decrease in BCAA catabolism activity was associated with low serum concentrations of leucine ketoacids. High serum BCAA (especially leucine) concentrations were associated with obesity and hyperinsulinemia in the males in our study, which is in line with earlier studies suggesting that BCAAs may augment insulin secretion from the pancreas in insulin-resistant states [38,39]. Recent studies in rat adipocytes have identified that BCAAs act both as triggers of insulin secretion and also as important players in the augmentation of insulin signaling through the mTOR (mammalian target of rapamycin) and phosphatidylinositol 3-kinase signaling pathways [56]. Thus, normal BCAA metabolism may be critical for the maintenance of an appropriate insulin response. We propose that reduced mitochondrial BCAA catabolism (possibly as a consequence of low mtDNA levels), inhibition of adipose tissue differentiation, increased liver fat accumulation, and stimulation of insulin secretion by high serum BCAA levels may explain part of the well-documented association between fatty liver and hyperinsulinemia during the development of insulin resistance syndrome [57].

An additional novel finding in our study was the down-regulation of several NAD^+^-dependent histone deacetylases or their associated proteins (e.g., *HDAC5, SIN3, SIRT1, SIRT3, and SIRT7*) in the fat tissue of the obese co-twins. Sirtuins (SIRTs) have been shown to mediate the response to caloric restriction, and their overexpression mimics this response in disease models (SIR2 in C. elegans), leading to an extended life span. Furthermore, they also regulate insulin-signaling and energy metabolic pathways [40]. In humans, *SIRT1* gene expression in skeletal muscle has been shown to increase in caloric restriction with or without exercise and in vitro by adiponectin treatment [58]. Our findings of the coordinated down-regulation of sirtuin expression in obese-human fat are, to our knowledge, first of its kind, and may be a consequence of decreased NAD synthesis and reduced energy utilization when abundant calories are available. Low NAD levels and increased NADH and nicotinamide levels could inhibit the sirtuins, thus initiating a cascade of events through numerous pathways that would (1) decrease transcription of genes for mitochondrial biogenesis and potentially reduce mtDNA copy number; and (2) decrease lipolysis through PPARs, thus promoting obesity [40].

The MZ co-twin control study represents perhaps the best controlled study design available in humans because of the complete or close match for genes, age, gender, and intrauterine and childhood environment. However, when used retrospectively as done here, it does not solve the problem of direction of causation. For this reason, we include some results from one obese study participant who had gained weight upon a follow-up visit. These data, although they essentially amount to a case report, do show convincingly a further pathological progression in the same measures identified as differing between the obese and non-obese twins. Despite the limited sample size, this, in addition to the trend for increased effects in twins with greater obesity discordance, lend support to the notion that the changes identified in this limited study sample are indeed sequelae, not proximal causes of obesity.

This study provides evidence for the contribution of lifestyle to the metabolic disturbances in obesity. However, it does not devalue the effects of genes. The fact that it was extremely difficult to find young, healthy MZ twins with large weight differences (14 twin pairs out of 2,453) itself suggests a genetic basis for obesity. The large differences in BMI within these most-discordant pairs may have arisen because of the presence of “environmental sensitivity/variability” genes that make these individuals particularly susceptible to environmental variation (such as diet and exercise), whereas other pairs who were concordant for BMI may carry alleles that render them relatively impervious to dietary or physical activity patterns. While the contribution of possible epigenetic changes [59] cannot be conclusively ruled out, they seem unlikely to explain this adult discordance, as all twins analyzed in the current study exhibited remarkably similar development of BMI until the onset of obesity in their early adulthood [23].

In summary, this study identified several changes in transcript profiles of adipose tissue early in acquired obesity by using the obesity-discordant MZ co-twin control design that allows full control for genomic sequence variations, a significant confounder in most studies comparing obese and non-obese participants. Our results highlight the metabolic plasticity and alterations of human adipose tissue. Our most critical findings were that, in the fat of obese co-twins compared to their non-obese counterparts, (1) mtDNA content was reduced, (2) mitochondrial energy production pathways were down-regulated along with adipocyte differentiation within subcutaneous adipose tissue, and (3) increase in ectopic fat deposition was subsequently increased. These events probably represent important drivers of insulin resistance, hyperinsulinemia, and accumulation of liver fat, pathognomonic characteristics of obesity acquired already in the young. It is likely that proper management of obesity, primarily by lifestyle changes, and perhaps with a new generation of therapies directed at several targets in mitochondrial biogenesis pathways, will correct these abnormalities and favorably modify the risk, course, and outcome of diabetes and cardiovascular diseases.

## Supporting Information

Figure S1 Sequence Variants in mtDNA Identified in the Most Discordant Twin Pair with a BMI Difference of 10.1 kg/m^2^
Columns 1 and 2 show variant position in reference sequence AC_000021.1 and corresponding reference nucleotide. Columns 3 to 6 show variant genotypes of the co-twins from leukocytes and fat. Heteroplasmy found at position 311 was present in both leukocytes and fat. Mitochondrial DNA sequences from blood leukocytes and fat samples were identical in all twin pairs sequenced (unpublished data).
(1.9 MB TIF)

Table S1 Pathways Up-regulated in the Obese Co-twins: Elaborated (100 KB DOC)

Table S2 Pathways Down-regulated in the Obese Co-twins: Elaborated (49 KB DOC)

Text S1 Supplemental Methods (117 KB DOC)

### Accession Numbers

The microarray data reported in this manuscript have been deposited in ArrayExpress (http://www.ebi.ac.uk/), accession number E-MEXP-1425.

## Abbreviations

BCAA: branched chain amino acid
BMI: body mass index
CRP: C-reactive protein
DEXA: dual-energy x-ray absorptiometry
fS-insulin: fasting serum insulin, GO, Gene Ontology
LDL: low-density lipoprotein
LPC: lysophosphatidylcholine
mtDNA: mitochondrial DNA
MZ: monozygotic
SD: standard deviation
