# Supplementary material for: Global Transcript Profiles of Fat in Monozygotic Twins Discordant for BMI: Pathways behind Acquired Obesity
Source: PLoS Med. 2008 Mar 11;5(3):e51. doi: 10.1371/journal.pmed.0050051 (PMC2265758; doi:10.1371/journal.pmed.0050051)
Supplement: Supplementary file 1 [file pmed.0050051.sd001.doc]

**Insulin sensitivity**

Whole body insulin sensitivity was determined by the euglycemic hyperinsulinemic clamp technique^1^. Two 18-gauge catheters (Venflon; Viggo-Spectramed, Helsingborg, Sweden) were inserted, one in an antecubital vein for infusion of insulin and glucose, and another retrogradely in a heated hand vein to obtain arterialised venous blood for measurement of glucose concentrations every 5 min and serum free insulin concentration every 30 min. Regular human insulin (Insulin Actrapid; Novo Nordisk, Denmark) was infused in a primed-continuous fashion. The rate of the continuous insulin infusion was 40 mU/m^2^·min (1 mU/kg⋅min) for 120 min. Normoglycaemia was maintained by adjusting the rate of a 20% glucose infusion based on plasma glucose measurements from arterialised venous blood every 5 min. Whole body insulin sensitivity (the M-value, expressed as mg/kg fat free mass) was determined from the glucose infusion rate after correction for changes in the glucose pool size^1^. Since hepatic glucose production is maximally suppressed in non-diabetic subjects already at an insulin concentration achieved during infusion of insulin at a rate of 0.5 mU/kg⋅min^2^, the M-value mostly reflects glucose uptake. Plasma glucose concentrations were measured in duplicate with the glucose oxidase method (Glucose Analyzer II; Beckman Instruments, Fullerton, CA, USA)^3^. Serum free insulin concentrations were determined with radioimmunoassay (Phadeseph Insulin RIA, Pharmacia & Upjohn Diagnostics, Uppsala, Sweden) after precipitation with polyethylene glycol^4^.

**Biochemical analyses**

Serum leptin concentrations were measured using enzyme-linked immunoassays (Quantikine R&D Systems, Minneapolis, MN, USA) and adiponectin concentrations using the ELISA kit from B-Bridge International (San Jose, CA, USA). High-sensitivity C-reactive protein (CRP) was measured using a sensitive double antibody sandwich ELISA with rabbit antihuman CRP and peroxidase conjugated rabbit anti-human CRP.

**Measurement of serum concentrations of leucine, isoleucine and valine**

25 µL water containing 40 µM α-amino butyric acid as internal standard, 5 µL water and 25 µL acetonitrile were added to 5 µL weighted serum sample and vortexed for 15 seconds and allowed to settle for 30 minutes. After centrifugation (10000 g 5 min.) 50 µL sample was withdrawn and dried under nitrogen. Dried sample was redissolved into 24 µL Waters AccQ Fluor borate buffer and 8 µL of AccQ-fluor reagent was added. 2 μL injection was done from this solution.

The instrument consisted of UPLC liquid chromatograph, Atlantis dC18 column (2.1x100 mm with 3 µm particles at 42^o^C) and M474 fluorescence detector (_ex._=250nm;_em._=395 nm). The eluent consisted of 140 mM triethylamine (pH 5.1) and acetonitrile. A complex gradient was run where acetonitrile concentration was increased from original 7% to 18% during 27 minutes. The system was controlled and data was treated with Empower software from Waters Inc. (Milford, MA, USA), which was also the supplier of the hardware employed.

**Determination of α-ketoacids**

The method is based on published method by T.Hayashi et al.^5^ based on the reaction of α-ketoacids with o-phenylenediamine to form UV- absorbing quinoxalinols.

5 µl serum sample was weighed, 5 µL of aqueous ketohexanoic acid (10 mg/L) was added as internal standard, 5 µL of water and 50 µL of 1% aqueous sulphosalisylic acid were then added. The solution was vortexed for 15 sec. and proteins were allowed to precipitate in ice-bath for 10 minutes. Solution was centrifuged (10000 g, 5 min). 25 µL of o-phenylenediamine was added to supernatant (2.66 g/L in 3M HCl) was added. Solution was heated at 80^o^C for 20 minutes, then cooled in ice bath and then extracted with ethyl acetate. Ethyl acetate extract was dried with Na_2_SO_4_ and dried with nitrogen. Finally it was dissolved in 25 µL 40% methanol/water.

The chromatographic system consisted of UPLC liquid chromatograph, BEHC18 column (1x50mm with 1.7 µm particles at 40^o^C) and PDA2996 diode array detector monitoring at 340 nm. The eluent was water:acetonitrile starting at 10% and after 2 minutes a linear gradient was run to 50% during 8 minutes. Injection volume was 2 µL. The system was controlled and data was treated with Empower software from Waters Inc. (Milford, MA, USA), which was also the supplier of the hardware employed.

**Total RNA preparation and array processing**

A needle aspiration biopsy of subcutaneous abdominal fat at the level of the umbilicus was taken under local anesthesia ^6^. The fat sample was immediately frozen and stored in liquid nitrogen until analysis. Part of the biopsy was treated with collagenase for isolation of fat cells for 30 minutes at 37ºC. From this sample, the mean diameter of 200 adipocytes was determined using a light microscope. Total RNA was prepared from frozen fat tissue (on average 250 mg) using the RNeasy Lipid Tissue Mini Kit (Qiagen) according to the manufacturer’s protocol. Quality of RNA was analyzed using the 2100 Bioanalyzer platform (Agilent Technologies). Two micrograms of total RNA were treated according to the conventional Affymetrix eukaryotic RNA labeling protocols (Affymetrix, Santa Clara, CA). 15 micrograms of biotin labeled cRNA were fragmented according Affymetrix eukaryotic sample protocol. Hybridization, staining and washing of the Affymetrix U133 Plus 2.0 chips were performed using the Affymetrix Fluidics Station 450 and Hybridization Oven 640 under standard conditions. Preprocessing of expression data was done using the GC-RMA algorithm. Prior to analysis, the expression values were co-twin normalized, which involved dividing the obese twin’s expression values with that of those of the thin co-twin in order to correct for the identical genetic back-ground.

**Pathway analysis**

In order to identify the affected biological pathways, which may not necessarily be defined by significant individual gene expression changes, a custom made non-parametric pathway analysis algorithm was employed using the gene annotation data from the GeneOntology Consortium. Briefly, the probe sets were ranked by the median fold change values between obese and lean twins to “upregulated” and “downregulated” vectors. The Affymetrix ProbeSetIDs were mapped to human genes using cross-references in the Ensembl database and the genes were queried for their GO annotations. The topology of the GO-tree (DAG tree) was fully utilized by enumerating all available routes towards the root of the GO tree and adding all encountered vertexes as GO annotations of the given gene. For detecting the affected GO gene groups (“pathways”), an iterative cumulative hypergeometric distribution p-value based calculation was used. The probability of the pathway being regulated in gene expression data is calculated for each occurrence of a gene belonging to the given pathway while iterating from up to down in the ranked list by:

$$p(j,k,t,n)=1-\sum_{c=0}^{k-1} \frac{\left(\begin{matrix} j \\ c \end{matrix}\right)\left(\begin{matrix} n-j \\ t-c \end{matrix}\right)}{\left(\begin{matrix} n \\ t \end{matrix}\right)}$$

Here *j*=absolute rank of the probeset/gene; *k*=rank of the probeset /gene amongst all the members of the pathway; *t*=total number of probeset /genes in the pathway; and *n*=total number of probeset/genes in the experiment. The objective is to find the optimum p-value for a set of genes which belong to the same annotated GO gene collection (maximal regulation for the pathway).

Further, the data was exhaustively permuted by randomizing the gene ranks for each GO category and an empirical p-value was interpreted from the distribution of 10,000 permutation cycles. A characteristic of the hierarchically structured GO trees is that gene sets located further down the tree get progressively larger, containing an increasing number of smaller gene sets and thus at the same time get progressively less descript. In order to focus on the more concise, biologically more relevant pathways a somewhat arbitrary cut-off number of 250 genes was chosen.

The mean-centroid value representing the activity of the whole regulated part of the pathway is calculated by normalizing the expression levels of the regulated genes in the pathway to a mean of zero and a variance of 1 across all individuals.

**Analyses of mitochondrial sequence and copy-number**

## Known mitochondrial DNA sequence variants were extracted from MITOMAP database ([**www.mitomap.org**](http://www.mitomap.org/)) and variant information was annotated to the selected reference sequence AC000021.1 (GI:58615662) from GenBank. PCR primers were selected and re-optimized among those presented by Sigurdsson et al ^7^. Sequencing primers were designed to avoid known variant positions using The PCR Suite ^8^. The mitochondrial genome was PCR amplified in two overlapping ~9 kb fragments. PCR amplification was performed using 20-30 ng of DNA, 14 pmol each primer, 200 μM dNTP 1,4 U of DyNAzyme EXT DNA polymerase in 1X DyNAzyme EXT buffer (Finnzymes). Thermocycling consisted of denaturation of DNA template in 94ºC for 2 min followed by 30 cycles of 94ºC for 20s, 60ºC for 30s and of 72ºC for 4 min (extended for 10 s / cycle) and final extension of 72º for 15 min. Correct amplification was verified by agarose gel electrophoresis. PCR products were ExoI / SAP purified and sequencing was performed with BigDye3.1 chemistry on an ABI 3730xl DNA Analyzer. Mitochondrial consensus sequences and sequence variants were determined with SeqScape Software v2.5 (Applied Biosystems). Oligonucleotide sequences used in PCR and sequencing are presented in the Appendix of Supplementary Methods (vide infra).

Mitochondrial DNA quantification

The ABI Prism 7000 Detection System Cycler was used to quantify mitochondrial DNA amount. The mitochondrial cytochrome b (Cytb) and the nuclear amyloid protein beta precursor protein (APP) were simultaneously amplified by quantitative real-time polymerase chain reaction (PCR) using the following primers:

**hCytb_F**	5’-GCCTGCCTGATCCTCCAAAT-3’

**hCytb_R**	5’-AAGGTAGCGGATGATTCAGCC-3’

**hAPP_F1**	5’-TGTGTGCTCTCCCAGGTCTA-3’

**hAPP_R1**	5’-CAGTTCTGGATGGTCACTGG-3’

For mtDNA and nuclear DNA quantification the following hybridization probes (Applied Biosystems UK) were used:

**h_cytB-FAM**	6-FAM 5’-CACCAGACGCCTCAACCGCCTT-3’ TAMRA

**h_APP_VIC**	VIC 5’-CCCTGAACTGCAGATCACCAATGTGGTAG-3’ TAMRA

Standard curves were constructed based on amplification of cloned plasmids containing the human APP or CytB gene. Standards were included in each run and used at concentrations of 10^3^-10^6^ copies.

The PCR reaction mixture (30µl) contained 25ng of DNA, 1xTaqMan Universal PCR Master Mix (Roche, Applied Biosystems), 900 nM of each primer and 250 nM of each probe. The PCR amplification consisted of a single enzyme activation step for 2 min at 50 ^o^C, denaturation for 10 min at 95 ^o^C, followed by 40 amplification cycles of 15 s at 95^o^C, and 1 min at 60^o^C. A single fluorescence acquisition was done at the end of each annealing step, and data was analyzed using the ABI Prism 7000 software (Applied Biosystems), at the exponential phase of the amplification. The efficiency of amplification in cytb and APP were confirmed to be equal within a sample, allowing accurate quantification. The ratio of mtDNA to nuclear DNA was calculated and used as a measure of mtDNA content and converted to percentage in each specimen.

Reference List

1.	DeFronzo,R.A., Tobin,J.D. & Andres,R. Glucose clamp technique: a method for quantifying insulin secretion and resistance. *Am. J. Physiol* **237**, E214-E223 (1979).

2.	Yki-Järvinen,H., Young,A.A., Lamkin,C. & Foley,J.E. Kinetics of glucose disposal in whole body and across the forearm in man. *J. Clin. Invest* **79**, 1713-1719 (1987).

3.	Kadish,A.H., Little,R.L. & Sternberg,J.C. A new and rapid method for the determination of glucose by measurement of rate of oxygen consumption. *Clin Chem* **14**, 116-131 (1968).

4.	Desbuquois,B. & Aurbach,G.D. Use of polyethylene glycol to separate free and antibody-bound peptide hormones in radioimmunoassays. *J. Clin. Endocrinol. Metab* **33**, 732-738 (1971).

5.	Hayashi,T., Tsuchiya,H., Todoriki,H. & Naruse,H. High-performance liquid chromatographic determination of alpha-keto acids in human urine and plasma. *Anal. Biochem.* **122**, 173-179 (1982).

6.	Yki-Järvinen,H., Nikkila,E.A., Kubo,K. & Foley,J.E. Assay of glucose transport in human fat cells obtained by needle biopsy. *Diabetologia* **29**, 287-290 (1986).

7.	Sigurdsson,S., Hedman,M., Sistonen,P., Sajantila,A. & Syvanen,A.C. A microarray system for genotyping 150 single nucleotide polymorphisms in the coding region of human mitochondrial DNA. *Genomics* **87**, 534-542 (2006).

8.	van Baren,M.J. & Heutink,P. The PCR suite. *Bioinformatics.* **20**, 591-593 (2004).

Appendix. Oligonucleotide sequences used in PCR and mitochondrial DNA sequencing

| **PCR primers** | **Position in AC000021.1** |
| --- | --- |
| CTACTCTACCATCTTTGCAGGC | 4496-4517 |
| CTGCTGCGAACAGAGTGG | 13204-13187 |
| CTCGAACTGACACTGAGCC | 12525-12543 |
| GGGTTGTACGGTAGAACTGC | 5062-5043 |
|  |  |
|  |  |
| **Sequencing primers** | **Position in AC000021.1** |
| GGGTTGTACGGTAGAACTGC | 5062-5043 |
| TTAAACCAAACCCAGCTACG | 4977-4996 |
| GATTAGGCGTAGGTAGAAGTAGAG | 5360-5337 |
| ACCAAGAGCCTTCAAAGCC | 5533-5551 |
| GTAACGACCACATCTACAACG | 6049-6069 |
| GAAAGTTAGATTTACGCCGATG | 7178-7157 |
| AGGAAAAGGGCATACAGGAC | 7716-7697 |
| TTAGGGGAATTAATTCTAGGACG | 8234-8212 |
| TTCCTTATCTGCTTCCTAGTCCT | 7679-7701 |
| CTACGGTCAATGCTCTGAAATC | 8161-8182 |
| TGGGTCATTATGTGTTGTCG | 9214-9195 |
| TCCTTAATCATTTTTATTGCCAC | 8746-8768 |
| ATCCAAGCCTACGTTTTCAC | 9151-9170 |
| TACCCTCCTACAAGCCTCAG | 9725-9744 |
| CGTCCCTTTCTCCATAAAATTC | 10202-10223 |
| TGCTAAAACTAATCGTCCCAAC | 10761-10782 |
| TCACAACACCCTAGGCTCAC | 11260-11279 |
| AGGATGGGGGGAATTAGG | 12395-12378 |
| CTTCTAGCAAGCCTCGCTAAC | 11837-11857 |
| TGGTGCAACTCCAAATAAAAG | 12314-12334 |
| CTGCTGCGAACAGAGTGG | 13204-13187 |
| CGCCTTAGCATGATTTATCC | 12888-12907 |
| CCCCATTCTATACCAACACC | 6584-6603 |
| AAATGTTGAGCCGTAGATGC | 9797-9778 |
| CTCGAACTGACACTGAGCC | 12525-12543 |
| TTCCTGCTACAACTATAGTGCTT | 13105-13083 |
| ACTCCCCTCAGCCATAGAAG | 13032-13051 |
| ATCTCATCGCTACCTCCCTG | 13571-13590 |
| CACAGCACCAAATCTCCAC | 14036-14054 |
| CTATTAAACCCATATAACCTCCC | 14511-14533 |
| CCTGAAACATCGGCATTATC | 15081-15100 |
| TTCCTATTCGCCTACACAATTC | 15566-15587 |
| TTCTTTCATGGGGAAGCAG | 16024-16042 |
| TCACCCTATTAACCACTCACG | 14-34 |
| GAGCCCGTCTAAACATTTTC | 630-611 |
| AGCTGTGGCTCGTAGTGTTC | 1157-1140 |
| AACCAAACCCCAAAGACAC | 550-568 |
| GATACCCCACTATGCTTAGCC | 1079-1099 |
| AGGAGACAAGTCGTAACATGG | 1550-1570 |
| TTAAATTTGCCCACAGAACC | 2048-2067 |
| GTACCCTAACCGTGCAAAGG | 2574-2593 |
| GAGTAATCCAGGTCGGTTTC | 3080-3099 |
| GGCCTCCTATTTATTCTAGCC | 3607-3627 |
| AAAACTTCCTACCACTCACC | 4178-4197 |
| CTACTCTACCATCTTTGCAGGC | 4496-4517 |
|  |  |
| Additional sequencing primers if more sequence coverage was required | |
| **Sequencing primers** | **Position in AC000021.1** |
| CTACTCTACCATCTTTGCAGGC | 4496-4517 |
| AACTAGTCAGTTGCCAAAGCC | 6152-6132 |
| TCCCCTATTCTCAGGCTACAC | 7094-7114 |
| ATGGGAGATTATTCCGAAGC | 6673-6652 |
| GAGGAGGTTAGTTGTGGCAA | 8781-8762 |
| TCATGGTAGGGGTAAAAGGAG | 10289-10269 |
| GTGGGTGGTTGTGTTGATTC | 10848-10830 |
| TTGGCTCAGGAGTTTGATAG | 11334-11315 |
| CACAGAGAGTTCTCCCAGTAGG | 11902-11881 |
| AGCGTTTAGAAGGGCTATTTG | 12954-12934 |
| AAGCGAGGTTGACCTGTTAG | 13646-13627 |
| AGGAAGTAAAGTTTAATTATGCC | 14102-14080 |
| TCTGAATTTTGGGGGAGG | 14545-14528 |
| GATATTTGGCCTCACGGG | 15162-15145 |
| GGATGGGGATTATTGCTAGG | 15665-15646 |
| ATACATAGCGGTTGTTGATGG | 16092-16072 |
| CTGCGACATAGGGTGCTC | 124-107 |
| TGTAAGTTGGGTGCTTTGTG | 1636-1617 |
| TGTCCAAAGAGCTGTTCCTC | 2124-2105 |
| AGACAGCTGAACCCTCGTG | 2658-2640 |
| GAAGGCGCTTTGTGAAGTAG | 3168-3149 |
| CAGGGCGTAGTTTGAGTTTG | 3693-3674 |
| TGGAGATTGTAATGGGTATGG | 4243-4223 |
| TGGGACTCAGAAGTGAAAGG | 4817-4798 |
| GGGTTGTACGGTAGAACTGC | 5062-5043 |
|  |  |
