# Supplementary material for: Global Transcript Profiles of Fat in Monozygotic Twins Discordant for BMI: Pathways behind Acquired Obesity
Source: PLoS Med. 2008 Mar 11;5(3):e51. doi: 10.1371/journal.pmed.0050051 (PMC2265758; doi:10.1371/journal.pmed.0050051)
Supplement: Supplementary file 2 [file pmed.0050051.st001.doc]

**Supplementary Table 1:** Pathways upregulated in the obese co-twins –elaborated

| **GO Category** | **Pathway Name** | **Nominal *P*** | **Empirical *P*** | **# of Genes in Pathway** | **Genes until optimum pathway composition reached** |
| --- | --- | --- | --- | --- | --- |
| GO:0019884 | Antigen processing and presentation of exogenous antigen | 2.99E-11 | 0.0001 | 21 | HLA-DQB1  HA25_HUMAN  HB21_HUMAN  HLA-DRB1  2B19_HUMAN  HLA-DRB5  FCER1G  NP_061984.2  HG2A_HUMAN  HA2Q_HUMAN  HLA-DPB1 |
|  |  |  |  |  |  |
| GO:0006954 | Inflammatory response | 1.16E-10 | 0.0001 | 241 | SPP1  IL1RN  CXCL9  CCL8  PLA2G7  CCL18  CCL5  THBS1  CCR1  AIF1  CXCL10  C163A_HUMAN  FCER1A  ITGB2  CCL2  C3AR1  FCER1G  PTGER3  TLR4  AOX1  CYBB |
|  |  |  |  |  |  |
| GO:0006959 | Humoral immune response | 1.31E-10 | 0.0001 | 169 | CD86  CCL18  C1S  KLRB1  C1QC  CLEC7A  TREM2  ENTPD1  ITGB2  IL7R  CCL2  LILRB2  C1QB  FCER1G  CFB  CLU  CYBB  NP_061984.2 |
|  |  |  |  |  |  |
| GO:0007626 | Locomotory behaviour | 1.00E-09 | 0.0001 | 177 | SPP1  CXCL9  CCL8  CCL18  UCHL1  CCL5  CCR1  CXCL10  ECGF1  CX3CR1  PTGD2_HUMAN  ITGB2  CCL2  EPHA4  C3AR1  FCER1G |
|  |  |  |  |  |  |
| GO:0016064 | Immunoglobulin mediated immune response | 1.06E-09 | 0.0001 | 123 | CCL18  C1S  KLRB1  C1QC  CLEC7A  ENTPD1  ITGB2  IL7R  LILRB2  C1QB  FCER1G  CLU  CYBB  NP_061984.2  HG2A_HUMAN  TNFSF13B  TLR8  CD53 |
|  |  |  |  |  |  |
| GO:0015629 | Actin cytoskeleton | 3.30E-09 | 0.0001 | 223 | ACTB  GSN  ACTR2  AIF1  CLIC5  FLNA  CORO1C  CLIC4  IQGAP1  SORBS1  LCP1  MARCKS  NP_878913.1  PTK9  CASK  MYO5A  MYOT  DBN1  ARPC5  MYO1D  ACTN1  FYB  CAPZB  IQGAP2  STK17B  TMOD3  CTNNA1  MYH10  BRPF1  CAPG  ACTR3  MYO6  SGPP1  RDX  IPP |
|  |  |  |  |  |  |
| GO:0019882 | Antigen presentation | 5.02E-09 | 0.0001 | 56 | HLA-DQB1  HA25_HUMAN  HB21_HUMAN  HLA-DRB1  2B19_HUMAN  HLA-DRB5  FCER1G  NP_061984.2  HG2A_HUMAN  HA2Q_HUMAN  IGLC1  HLA-DPB1  Q5STC4_HUMAN  RELB  IGHG1  NM_005931.2 |
|  |  |  |  |  |  |
| GO:0006935 | Chemotaxis | 1.26E-08 | 0.0001 | 130 | SPP1  CXCL9  CCL8  CCL18  CCL5  CCR1  CXCL10  ECGF1  CX3CR1  ITGB2  CCL2  C3AR1  FCER1G |
|  |  |  |  |  |  |
| GO:0042330 | Taxis | 1.26E-08 | 0.0001 | 130 | SPP1  CXCL9  CCL8  CCL18  CCL5  CCR1  CXCL10  ECGF1  CX3CR1  ITGB2  CCL2  C3AR1  FCER1G |
|  |  |  |  |  |  |
| GO:0006968 | Cellular defense response | 5.88E-07 | 0.0001 | 118 | SPP1  CXCL9  CD86  CCL5  LBP  CX3CR1  LILRB2  TRDV2  C3AR1  FCER1G  TLR4  TYROBP |
|  |  |  |  |  |  |
| GO:0019838 | Growth factor binding | 1.57E-06 | 0.0002 | 57 | CTGF  WISP2  LTBP2  IL7R  IL10RA  NTRK2  IL6ST  IL1R2  CYR61  LTBP3  NOV |
|  |  |  |  |  |  |
| GO:0042591 | Antigen processing and presentation of exogenous peptide antigen via MHC class II | 1.86 E-06 | 0.0001 | 8 | HLA-DQB1  FCER1G  NP_061984.2  HG2A_HUMAN  Q5STC4_HUMAN |
|  |  |  |  |  |  |
| GO:0016197 | Endosome transport | 1.99 E-06 | 0.0002 | 33 | FLNA  EEA1  VPS35  EHD1  RAB6IP2  M6PR  PAMCI  RHOB  VPS26B  SQSTM1 |
|  |  |  |  |  |  |
| GO:0006887 | Exocytosis | 2.17 E-06 | 0.0001 | 80 | CCL8  CCL5  ADSV_HUMAN  VAMP8  Q9Y6V0-3  FCER1A  EXOC7  XP_942255.1  YKT6  UN13C_HUMAN |
|  |  |  |  |  |  |
| GO:0051240 | Positive regulation of multicellular organismal process | 2.34 E-06 | 0.0003 | 110 | ? |
|  |  |  |  |  |  |
| GO:0005178 | Integrin binding | 2.52 E-06 | 0.0002 | 39 | SPP1  EGFL6  CTGF |
|  |  |  |  |  |  |
| GO:0009897 | External side of plasma membrane | 4.06 E-06 | 0.0001 | 52 | CD86  HLA-DQB1  FCER1A  IL7R  NRCAM  FCER1G  NP_061984.2  HG2A_HUMAN  CD44 |
|  |  |  |  |  |  |
| GO:0005125 | Cytokine activity | 4.30 E-06 | 0.0003 | 220 | SPP1  IL1RN  CXCL9  ANGPT2  CCL8  CCL18  CCL5  ANGP1_HUMAN  CXCL10  ECGF1  IRAK1 |
|  |  |  |  |  |  |
| GO:0008009 | Chemokine activity | 4.87 E-06 | 0.0002 | 42 | CXCL9  CCL8  CCL18  CCL5  CXCL10 |
|  |  |  |  |  |  |
| GO:0042379 | Chemokine receptor binding | 5.49 E-06 | 0.0001 | 43 | CXCL9  CCL8  CCL18  CCL5  CXCL10 |

Nineteen pathways with less than 250 members were identified as significantly upregulated in the adipose tissue of the obese co-twins following co-twin normalization of global expression values. Results are ranked according to significance (empirical P-values interpreted from the distribution of 10,000 permutation cycles).
