# Supplementary material for: Global Transcript Profiles of Fat in Monozygotic Twins Discordant for BMI: Pathways behind Acquired Obesity
Source: PLoS Med. 2008 Mar 11;5(3):e51. doi: 10.1371/journal.pmed.0050051 (PMC2265758; doi:10.1371/journal.pmed.0050051)
Supplement: Supplementary file 3 [file pmed.0050051.st002.doc]

**Supplementary Table 2:** Pathways downregulated in the obese co-twins –elaborated

| **GO Category** | **Pathway Name** | **Nominal *P*** | **Empirical *P*** | **# of Genes in Pathway** | **Genes until optimum pathway composition reached** |
| --- | --- | --- | --- | --- | --- |
| GO:0009081 | Branched chain family amino acid metabolism | 5.42E-08 | 0.0001 | 11 | ALDH6A1  AUH  BCKDHB  HIBADH  MCCC2  MCCC1  ACAD8  BCAT2 |
|  |  |  |  |  |  |
| GO:0045892 | Negative regulation of transcription | 4.81E-07 | 0.0001 | 182 | ORC2L  SIN3A  HDAC5  SIRT3  SUV39H1  ZBTB16  LDB1  SIN3B  PPARG  CDKN1C  RYBP  BHLHB2  JARID1B  TWIST1  HIC2  SNAI2  BCOR  MXD4  FALZ_HUMAN  NP_001012661.1  DAXX  NP_004873.3  ARID4A  SOX9  MXI1  SIRT1  PURA  HIPK2  ING4_HUMAN  ZNF202  TBX2  PIAS4  PGEA1  SMARCC2  SIRT7  ZNF496  ZNF177  TRIM66  KLF3  MDM4  MLXIPL |
|  |  |  |  |  |  |
| GO:0016885 | Ligase activity, forming carbon-carbon bonds | 2.21E-06 | 0.0001 | 7 | ACACB  PCCA  MCCC2  MCCC1  PCCB  PC  ACACA |
|  |  |  |  |  |  |
| GO:009083 | Branched chain family amino acid catabolism | 6.26E-06 | 0.0002 | 9 | AUH  BCKDHB  MCCC2  MCCC1  ACAD8  BCAT2 |
|  |  |  |  |  |  |
| GO:0016421 | CoA carboxylase activity | 7.11E-06 | 0.0002 | 6 | ACACB  PCCA  MCCC2  MCCC1  PCCB |
|  |  |  |  |  |  |
| GO:0006767 | Water-soluble vitamin metabolism | 8.29E-06 | 0.0004 | 38 | NMNAT2  NMNAT3  PCCA  PBEF1  C9orf95  DCXR  TMLHE  HIBADH |
|  |  |  |  |  |  |
| GO:0007517 | Muscle development | 9.97E-06 | 0.0005 | 156 | SIX4  SVIL  IGFBP3  MKX  LARGE  HDAC5  IGF1  CSRP2  POU6F1 |

Seven pathways with less than 250 members were identified as significantly downregulated in the adipose tissue of the obese co-twins following co-twin normalization of global expression values. Results are ranked according to significance (empirical P-values interpreted from the distribution of 10,000 permutation cycles).
